# Restoring Glutathione Homeostasis in Glycation-Related Eye Diseases: Mechanistic Insights and Therapeutic Interventions Beyond VEGF Inhibition

**DOI:** 10.3390/antiox14060731

**Published:** 2025-06-14

**Authors:** Yong Chool Boo

**Affiliations:** 1 Department of Molecular Medicine, School of Medicine, Kyungpook National University, 680 Gukchaebosang-ro, Jung-gu, Daegu 41944, Republic of Korea; ycboo@knu.ac.kr; Tel.: +82-53-420-4946; 2 Department of Biomedical Science, BK21 Plus KNU Biomedical Convergence Program, The Graduate School, Kyungpook National University, Daegu 41944, Republic of Korea; 3 Cell and Matrix Research Institute, Kyungpook National University, Daegu 41944, Republic of Korea

**Keywords:** advanced glycation end-products, glutathione, oxidative stress, cataracts, diabetic retinopathy, age-related macular degeneration, aldose reductase inhibitors, glyoxalase, redox homeostasis

## Abstract

Advanced glycation end-products (AGEs) and oxidative stress are recognized as central contributors to the pathogenesis of age-related or diabetic cataracts, diabetic retinopathy (DR), and age-related macular degeneration (AMD). These glycation-related diseases are characterized by impaired redox balance and decreased glutathione (GSH) levels. This review aims to examine the mechanistic links between AGEs and GSH depletion across ocular tissues by integrating in vitro, ex vivo, in vivo, and clinical studies relevant to this topic. The multiple levels of evidence highlight GSH homeostasis as both a biomarker and therapeutic target in glycation-related ocular disorders. Therapeutic strategies aimed at restoring GSH homeostasis under glycation stress are categorized into four mechanistic domains: (I) promoting GSH supply and synthesis, (II) enhancing GSH recycling, (III) mitigating glycation stress, and (IV) reducing oxidative and nitrosative stress. Most of these strategies have been explored via different approaches, and experimental findings with various interventions have shown promise in restoring GSH balance and mitigating AGE-induced damage. A pathological link between GSH depletion and vascular endothelial growth factor (VEGF) overexpression is observed in DR and wet AMD. GSH-centered interventions act upstream to modulate redox homeostasis while anti-VEGF therapies target downstream angiogenesis. This study supports the rationale for a dual-targeting strategy that combines redox-based interventions with VEGF inhibition in glycation-related ocular diseases.

## 1. Introduction

Ocular diseases, such as age-related or diabetic cataracts, diabetic retinopathy (DR), and age-related macular degeneration (AMD), are major causes of vision impairment and blindness worldwide [1,2]. While their etiologies are multifactorial, increasing evidence implicates oxidative stress and metabolic dysfunction as central contributors to their pathogenesis [3]. Among the biochemical culprits, advanced glycation end-products (AGEs) have emerged as key players in the progression of age- and diabetes-related eye diseases [4].

AGEs are formed through the non-enzymatic glycation and oxidation of proteins, lipids, and nucleic acids, particularly under hyperglycemic and oxidative conditions [5,6]. These irreversible modifications disrupt cellular function by altering protein structure, promoting inflammation, and inducing oxidative stress [7,8,9]. In ocular tissues, AGEs accumulate in long-lived proteins of the lens and retina, where they can initiate or exacerbate degenerative processes [10].

One critical target of glycation and oxidation-induced eye disease is glutathione (GSH), a tripeptide composed of glutamate, cysteine, and glycine [11]. GSH not only scavenges reactive oxygen species (ROS), reactive nitrogen species (RNS), and reactive carbonyl species (RCS) but also participates in enzymatic detoxification pathways, such as those involving glutathione peroxidase (GPx) and glyoxalase (GLO) systems [12,13]. Dysregulation of GSH homeostasis—either by direct oxidative depletion or by AGE-mediated inactivation of related enzymes—compromises the antioxidant defense of the eye, making it more susceptible to glyco-oxidative damage [14,15].

Several previous review articles have examined the role of oxidative stress and AGEs in diabetes-related complications, including ocular pathologies. Pescosolido et al. reviewed cataractogenesis in the aging lens, noting AGE accumulation and declined GSH levels [16]. Babel and Dandekar provided a broad overview of oxidative stress pathways in diabetic complications, including systemic AGE-GSH interactions [17]. Fan et al. emphasized persistent gaps in our understanding of glutathione dynamics in the ocular lens, underlining the need for targeted strategies to preserve GSH homeostasis [18]. Lim et al. reported that GSH levels in the lens follow a circadian pattern, with maximum antioxidant capacity aligned with core clock gene activity during the early active phase [19]. Despite these contributions, these existing reviews have not comprehensively examined GSH homeostasis across diverse ocular conditions, nor have they emphasized therapeutic strategies aimed at restoring GSH balance in the context of glycation stress.

The present review aims to examine the interplay between AGEs and GSH homeostasis in ocular diseases. Section 2 and Section 3 provide a general background on the cellular and molecular aspects of glycation and GSH homeostasis, respectively. Section 4 summarizes key findings from in vitro, ex vivo, in vivo, and clinical studies that investigated alterations in GSH levels and redox balance under glycation stress in ocular tissues. Section 5 discusses mechanistic insights and therapeutic strategies, including emerging approaches to restore GSH levels and mitigate glycation-related ocular damage. This review highlights the therapeutic importance of maintaining redox homeostasis in ocular pathologies by identifying critical patterns and promising interventions. It is hoped that this work will advance the understanding of GSH homeostasis as both a biomarker and a therapeutic target in glycation-related eye diseases and foster the development of targeted redox-based therapeutic strategies.

## 2. Cellular and Molecular Aspects of Glycation

This section introduces the mechanisms of glycation stress, the characteristics of AGEs, and the enzymes involved in glycation-related metabolism.

### 2.1. Glycation Stress

Glycation stress refers to a cellular condition resulting from the accumulation of early and advanced glycation products formed through non-enzymatic reactions between RCS and nucleophilic sites in proteins, lipids, and DNA [5,6,20]. RCS, such as methylglyoxal (MG) and glyoxal (GO), are generated as by-products of glycolysis, lipid peroxidation, and the autoxidation of sugars. Their accumulation is accelerated under hyperglycemic and oxidative conditions.

Initially, a reducing sugar reacts with a free amino group of a protein to form a Schiff base, which subsequently undergoes rearrangement into more stable Amadori products and Heyns products [7,21]. Over time, these early glycation products further degrade through oxidative and dehydration processes, leading to the generation of highly reactive intermediates and AGEs [22,23]. Hyperglycemia, oxidative stress, chronic inflammation, and aging are major factors that accelerate glycation reactions in vivo [24,25,26,27]. The biological consequences of glycation stress include loss of protein function, activation of inflammatory pathways, increased cellular apoptosis, and structural alterations in the extracellular matrix. Glycation and subsequent AGE accumulation contribute to the pathogenesis of various metabolic, cardiovascular, neurodegenerative, ocular, and age-related diseases [28].

### 2.2. AGEs

AGEs represent a heterogeneous group of complex, stable, and often irreversible adducts formed through prolonged glycation processes [7,29]. Table 1 summarizes various types of AGEs classified by precursor substrates and specific molecular target sites, including amino acid residues (e.g., lysine, arginine, and cysteine) of proteins, collagen, nucleic acids, and lipids.

AGEs can form either crosslinking structures between proteins or non-crosslinking adducts, depending on the reacting molecules and environmental conditions [35]. Crosslinking AGEs, such as glucosepane and pentosidine, form covalent bonds between amino acid residues (typically lysine and arginine) on long-lived proteins like collagen. Non-crosslinking AGEs, such as *N*^ε^-(carboxymethyl)lysine (CML) and *N*^ε^-(carboxyethyl)lysine (CEL), modify individual amino acid residues without forming crosslinks. Cellular responses to AGEs are primarily mediated by specific receptors, including the receptor for advanced glycation end-products (RAGE) and other advanced glycation end-product receptor (AGE-R) family members (AGE-R1, AGE-R2, and AGE-R3) [36]. Activation of RAGE signaling triggers downstream pathways such as nuclear factor kappa-light-chain-enhancer of activated B cells (NF-κB) activation, leading to the release of pro-inflammatory cytokines and the amplification of oxidative stress, thereby perpetuating tissue damage [37,38].

RAGE activation triggers NF-κB signaling, leading to pro-inflammatory cytokine release (e.g., tumor necrosis factor (TNF)-α and interleukin (IL)-6), which in turn can induce autophagy [39]. This self-protective process facilitates the degradation of intracellular AGEs and restores redox homeostasis. However, in chronic diseases like diabetes, sustained inflammation often fails to promote regeneration, contributing to prolonged tissue damage and dysfunction.

### 2.3. Glycation-Related Enzymes

Several enzymes regulate the levels of RCS and glycation products, thereby modulating glycation stress and protecting cellular integrity. The GLO system, comprising GLO1 and GLO2, plays a central role in detoxifying RCS, such as MG and GO [12,40]. This system utilizes GSH to catalyze the conversion of these reactive aldehydes into less harmful products, namely lactate and glycolate, thereby preventing the accumulation of AGEs. In addition, aldehyde dehydrogenase (ALDH) contributes to the detoxification of reactive aldehydes by catalyzing their oxidation into less reactive carboxylic acids [41]. Aldo-keto reductase (AKR) represents a broad superfamily of enzymes that catalyze the NADPH-dependent reduction of diverse carbonyl compounds, including MG and other reactive aldehydes [42]. Through this mechanism, AKR complements other detoxification pathways and supports the maintenance of cellular redox homeostasis.

Among the AKR family, aldose reductase (AR)—encoded by the AKR1B1 gene—is a specialized NADPH-dependent enzyme that primarily catalyzes the reduction of glucose to sorbitol, initiating the polyol pathway [43]. AR plays a dual role under glycation stress: while it may contribute to the reduction of reactive dicarbonyl compounds such as MG and GO, its primary function involves glucose metabolism. Under hyperglycemic conditions, its hyperactivation promotes sorbitol accumulation, osmotic stress, and subsequent lens damage, particularly in diabetic settings. Excessive activation of AR leads to substantial NADPH consumption, thereby limiting its availability for the glutathione reductase (GR)-mediated regeneration of GSH. Additionally, fructose produced via sorbitol dehydrogenase (SDH) in the polyol pathway undergoes glycation reactions at a substantially higher rate than glucose, owing to its greater proportion of open-chain form and the increased electrophilicity of its carbonyl group [44]. Consequently, the hyperactivation of AR not only exacerbates osmotic stress but also intensifies glycation stress, thereby accelerating the accumulation of AGEs [45].

Fructosamine-3-kinase (FR3K) acts at a later stage of the glycation pathway by phosphorylating Amadori products [46]. This modification promotes the degradation of early glycation intermediates, thereby reducing the formation of stable AGEs. Moreover, protein deglycase DJ-1 (Parkinsonism-associated deglycase 7, PARK7) protects cells through antioxidant and deglycation mechanisms [47]. Acting as a redox sensor via the oxidation of its conserved cysteine^106^ residue, DJ-1 promotes the nuclear translocation of nuclear factor erythroid 2-related factor 2 (Nrf2), thereby enhancing the expression of antioxidant enzymes. It also translocates to mitochondria to preserve complex I activity and suppress mitochondrial ROS production. In parallel, DJ-1 reverses early glycation adducts induced by RCS, contributing to the maintenance of proteostasis [48].

## 3. Cellular and Molecular Aspects of GSH Homeostasis

GSH is the most abundant intracellular thiol and plays crucial roles in cellular defense against oxidative stress, the detoxification of xenobiotics, and the regulation of redox-sensitive signaling pathways [49,50]. This section outlines the homeostasis of GSH, including its synthesis, degradation, and redox cycling, and discusses how GSH metabolism intersects with glycation processes to modulate cellular stress responses.

### 3.1. GSH Biosynthesis and Degradation

The cystine/glutamate antiporter xCT imports cystine in exchange for glutamate, supporting cysteine availability for GSH biosynthesis in cells [51]. The biosynthesis of GSH occurs in two sequential ATP-dependent enzymatic steps [52]. γ-Glutamylcysteine ligase (GCL) catalyzes the formation of γ-glutamylcysteine from glutamate and cysteine, representing the rate-limiting step. Glutathione synthetase (GS) then adds glycine to form GSH.

GSH degradation occurs through distinct pathways depending on its cellular location. Extracellular GSH is sequentially processed via the γ-glutamyl cycle, where γ-glutamyl transpeptidase (GGT) initiates GSH breakdown by transferring the γ-glutamyl group to acceptor amino acids, facilitating the uptake of precursor amino acids for intracellular GSH resynthesis [53]. The γ-glutamyl amino acids generated are subsequently converted into 5-oxoproline and free amino acids by γ-glutamylcyclotransferase (GGCT), further contributing to amino acid recycling [54]. In contrast, intracellular GSH is directly degraded by ChaC1 (glutathione-specific γ-glutamylcyclotransferase 1), particularly under endoplasmic reticulum (ER) stress conditions, producing 5-oxoproline and cysteinyl-glycine dipeptides [55]. The 5-oxoproline is then converted back to glutamate by 5-oxoprolinase, while cysteinyl-glycine is hydrolyzed by dipeptidases to release cysteine and glycine, both of which can be reutilized for de novo GSH synthesis [56].

### 3.2. GSH-Mediated Antioxidant Defense and Redox Regulation

GSH plays a central role in cellular antioxidant defense through both enzymatic and non-enzymatic mechanisms. Table 2 summarizes the key enzymatic and non-enzymatic reactions involving GSH, including the relevant enzymes and functional roles.

GPx catalyzes the reduction of hydrogen peroxide and lipid hydroperoxides using GSH, resulting in glutathione disulfide (GSSG) formation [57]. Subsequently, GR regenerates GSH from GSSG using NADPH [58]. Efficient operation of the GSH redox cycle is critical for maintaining the intracellular redox environment and protecting against oxidative stress associated with aging and disease [13].

Glutaredoxin (Grx, also referred to as thioltransferase or TTase) is a small redox enzyme that catalyzes the reversible *S*-glutathionylation and deglutathionylation of protein thiols [59,60,61]. This post-translational modification regulates redox signaling and protects cysteine residues from irreversible oxidative damage. Grx also functions as a GSH-dependent reductase, reducing both protein–glutathione mixed disulfides and interprotein disulfide bonds using GSH as an electron donor. This reaction is coupled with the GR reaction, constituting a tight redox cycle that maintains thiol homeostasis and protects cells from oxidative stress.

Glutathione *S*-transferase (GST) mediates the conjugation of GSH to electrophilic compounds, aiding in the detoxification of xenobiotics and endogenous toxicants [62]. Multidrug resistance-associated protein (MRP) 1, MRP1, MRP2, and MRP4 are involved in transporting GSH conjugates and other GSH-bound metabolites out of cells, further supporting cellular detoxification and GSH homeostasis [63].

GSH also functions through non-enzymatic mechanisms by directly scavenging a broad spectrum of ROS, such as hydroxyl radicals (^•^OH), superoxide anion radicals (O_2_^•−^), and singlet oxygen (^1^O_2_) [64]. Superoxide dismutase (SOD) effectively dismutates O_2_^•−^ to hydrogen peroxide (H_2_O_2_) [65]. The resulting H_2_O_2_ is subsequently detoxified into water by catalase (CAT), GPx, or peroxiredoxin (Prx) [66]. Moreover, GSH functions as a cofactor in both non-enzymatic and enzymatic (via Grx) pathways for the reduction of dehydroascorbate (DHA) back to ascorbate in human cells [67]. Restored ascorbate plays a vital role in scavenging ROS, regenerating other antioxidants, such as vitamin E, and supporting key biosynthetic functions [68,69].

Additionally, GSH neutralizes RNS, including nitric oxide (NO^•^) and peroxynitrite (ONOO^−^), with the latter being generated from the reaction of NO^•^ with O_2_^•−^ [70]. The *S*-nitrothiol derivative, nitrosoglutathione (GSNO), formed through the reaction of GSH and NO^•^, is further metabolized by NADH-dependent GSNO reductase (GSNOR) to an unstable intermediate [GSNHOH], which reacts with GSH, producing GSSG and hydroxylamine [71]. These processes help control nitrosative stress and maintain redox homeostasis.

GSH reacts non-enzymatically and reversibly with reactive aldehydes such as MG and GO to form hemithioacetals in solution [12]. Contrary to previous assumptions, hemithioacetals are not freely circulating substrates for GLO1. Instead, GSH and the reactive aldehyde bind sequentially within the active site of GLO1, where the hemithioacetal intermediate is formed in situ and immediately converted into *S*-lactoylglutathione or *S*-glycolylglutathione. These thioester intermediates are subsequently hydrolyzed by GLO2, producing lactate or glycolate, with concomitant regeneration of GSH [40].

**Figure 1:**
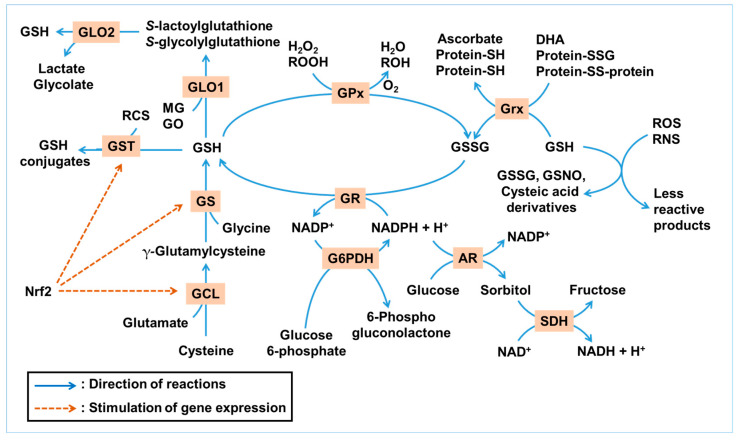
Glutathione (GSH)-centered antioxidant and antiglycation network. This illustration summarizes the antioxidant and antiglycation roles of GSH under metabolic stress. GSH can directly react non-enzymatically with a range of reactive oxygen species (ROS) and reactive nitrogen species (RNS), such as hydroxyl radicals (^•^OH), nitric oxide (^•^NO), and peroxynitrite (ONOO^−^), resulting in its conversion into GSSG, GSNO, or cysteic acid derivatives, while neutralizing ROS/RNS into less reactive products. Enzymatically, GSH serves as a substrate for glutathione peroxidase (GPx) to reduce hydrogen peroxide (H_2_O_2_) and lipid hydroperoxides (ROOH), and for glutaredoxin (Grx) to reduce protein disulfides and dehydroascorbate (DHA). GSH is regenerated from its oxidized form, GSSG, by glutathione reductase (GR) using NADPH. NADPH is mainly regenerated through glucose 6-phosphate dehydrogenase (G6PDH), with contributions from other NADPH-generating enzymes. The GLO system utilizes GSH to detoxify methylglyoxal (MG) and glyoxal (GO). Glutathione *S*-transferase (GST) conjugates GSH to various electrophilic xenobiotics and reactive carbonyl species (RCS). GSH is synthesized de novo via glutamate–cysteine ligase (GCL) and glutathione synthetase (GS) in two ATP-dependent steps. These enzymes are transcriptionally regulated by nuclear factor erythroid 2-related factor 2 (Nrf2), a master antioxidant response factor. The polyol pathway, initiated by aldose reductase (AR) and followed by sorbitol dehydrogenase (SDH), consumes NADPH during glucose-to-sorbitol conversion, reducing NADPH availability for GSH regeneration. Collectively, this network demonstrates how redox and carbonyl homeostasis in ocular tissues are maintained through a tightly regulated, NADPH-dependent enzymatic system centered on GSH. Blue arrows indicate the directionality of reactions, whereas brown dashed arrows denote the activation of gene expression.

Figure 1 illustrates the GSH-centered redox cycle and its integration with enzymatic pathways that detoxify ROS, RNS, and RCS. This network highlights the interplay between GSH synthesis, utilization, and regeneration under oxidative and glycation stress.

## 4. GSH Homeostasis in Glycation-Related Eye Diseases

This section provides an integrated overview of findings from in vitro, ex vivo, in vivo, and clinical studies, exploring the impact of glycation stress on GSH homeostasis and therapeutic interventions aimed at preserving or restoring redox balance in ocular tissues. The literature reviewed in this section was primarily retrieved through PubMed (https://pubmed.ncbi.nlm.nih.gov/, accessed on 26 April 2025) and Scopus (https://www.scopus.com/, accessed on 26 April 2025) using combinations of keywords, such as ‘advanced glycation end-products (AGEs)’, ‘glutathione’, ‘eyes’, ‘ocular’, ‘oxidative stress’, ‘diabetic’, ‘cataracts’, ‘retinopathy’, and ‘age-related macular degeneration (AMD)’. Relevant in vitro, ex vivo, in vivo, and clinical studies published from 1990 to March 2025 were included.

### 4.1. In Vitro Studies

Table 3 summarizes studies using in vitro cell culture models to investigate the effects of AGEs or interventions on GSH levels and associated biomarkers in eye-related experimental systems.

AGEs significantly impair antioxidant defenses in ocular cell types, thereby contributing to cellular dysfunction and the development of several eye diseases. Paget et al. observed that exposure to AGE-modified proteins led to divergent effects on CAT and SOD activity depending on the cell type, with pericytes showing increased antioxidant enzyme activity and endothelial cells showing no significant change [72]. These discrepancies highlight the cell-specific susceptibility to AGE-induced oxidative damage and provide insight into early pericyte dropout in DR. Shin et al. reported that glycation induced by high glucose concentrations (50–100 mM) decreased the viability of human lens epithelial cells (HLE-B3) and inactivated key antioxidant enzymes, accompanied by increased lipid peroxidation and DNA damage, suggesting a pro-oxidant shift in redox balance [74].

Several studies highlighted GSH’s central role in mitigating oxidative stress triggered by AGEs. Pigment epithelium-derived factor (PEDF) protected pericytes by decreasing ROS via the upregulation of GSH and SOD, while inhibiting caspase-3 activation through the Src pathway [76]. *O*-linked *N*-acetylglucosamine is reversibly added to intracellular proteins by *O*-linked *N*-acetylglucosamine transferase (OGT) and removed by *O-linked N-acetylglucosaminidase* (OGA). In human retinal microvascular endothelial cells, inhibition of OGA by PUGNAc reduced GO-induced ROS and enhanced antioxidant defenses, whereas OGT knockdown using siRNA elevated ROS levels and diminished antioxidant responses [79]. Liu et al. examined the function of TTase in human lens epithelial cells exposed to high glucose and advanced glycation end-products [85]. They found that oxidative stress induced by AGEs decreased CAT and SOD activities and increased the ratio of GSSG to total GSH, suggesting GSH depletion. TTase upregulation was associated with improved redox status, while TTase knockdown exacerbated oxidative damage, highlighting the enzyme’s role in maintaining GSH redox balance.

Numerous bioactive agents and novel compounds demonstrated the potential to prevent or reverse AGE-induced ocular damage through the modulation of oxidative pathways, often restoring GSH levels. Lehman and Ortwerth tested various inhibitors of AGE-associated protein crosslinking, including sulfur-containing compounds like cysteine and GSH, which showed effective inhibition of protein crosslinking processes relevant to ocular tissues [73]. In studies focusing on ocular protein stability, Mantha et al. found that antioxidants like GSH were essential in limiting AGE formation and protein photodegradation under light exposure, reinforcing the significance of GSH in maintaining protein integrity within the eye [83]. Abu-Kheit et al. studied *S*-allylmercapto-*N*-acetylcysteine, an antioxidant that increased GSH levels and decreased ROS in AGE-treated bone marrow stromal cells. Although this investigation centered on bone tissue, the study highlighted the systemic antioxidant properties of the compound, suggesting its potential relevance in mitigating AGE-related ocular disorders as well [86].

Fu et al. explored the protective role of berberine, a natural alkaloid compound, on human retinal Müller cells exposed to highly oxidized glycated low-density lipoprotein [81]. They demonstrated that berberine significantly reduced oxidative stress, autophagy, and apoptosis by enhancing adenosine monophosphate-activated protein kinase (AMPK) pathway activity, which included modulation of the GSH-related antioxidant responses. Natural phenolic compounds, such as (−)-epigallocatechin gallate (EGCG), phloretin, 6-shogaol, and 6-gingerol, protected retinal epithelial cells from MGO-induced cytotoxicity by activating Nrf2 and increasing heme oxygenase-1 (HO-1) and GSH expression [80]. Similarly, Wang et al. found that polyphenols like quercetin and cyanidin-3-glucoside not only suppressed AGE-related oxidative stress and photodegradation of a retinal fluorophore A2E (*N*-retinylidene-*N*-retinylethanolamine), but also preserved intracellular GSH levels in retinal pigment epithelial cells [82]. Jeon et al. further showed that treatment with caffeic acid significantly decreased AGE-induced oxidative stress and restored GSH levels, suggesting protective effects via β-catenin pathway inhibition [84]. Similarly, moscatilin alleviated oxidative stress in Müller glial cells by inhibiting p38-mitogen-activated protein kinase (MAPK)/c-Jun *N*-terminal kinase (JNK) and NF-κB signaling, thereby restoring GSH homeostasis and reducing inflammation [88]. *Peperomia pellucida* extract reduced AGE- and glucose-induced oxidative stress in retinal pigment epithelial cells by increasing GPx expression and modulating NF-κB and peroxisome proliferator-activated receptor gamma (PPAR-γ) signaling pathways [87].

AR inhibitors have been developed as therapeutic agents aimed at restoring intracellular NADPH levels, preserving antioxidant capacity, and mitigating AGE formation [89]. Papastavrou et al. introduced a new class of AR inhibitors with a 1-hydroxypyrazole scaffold, showing improved inhibitory activity and pharmacological profiles, which may complement antioxidant defenses in diabetic ocular pathologies [77]. In addition, silica-based CeCl_3_ nanoparticles inhibited the glycation of α-crystallin and enhanced chaperone activity while restoring GSH levels under H_2_O_2_ stress in lens epithelial cells [78].

The in vitro evidence highlights the crucial role of GSH in defending against AGE-induced oxidative damage across various ocular cell types. AGEs disrupt GSH homeostasis either directly via oxidative mechanisms or indirectly by inhibiting related enzymatic pathways. Therapeutic strategies that restore or enhance GSH levels—through synthetic precursors, natural compounds, enzymatic modulation, or nanomaterial-based delivery—represent promising avenues for mitigating glycation-associated ocular pathologies.

### 4.2. Ex Vivo Studies

Table 4 summarizes studies using ex vivo organ culture models, such as isolated lenses or retinal tissues, to assess the effects of AGEs or interventions on GSH levels and associated biomarkers.

Ex vivo lens culture models demonstrated that glycation inducers such as glucose or AGE precursors led to reductions in GSH and increases in AGE accumulation and lens stiffness or opacity. A study analyzing the optical properties of lenses exposed to high glucose found that glucose-induced opacity in human lenses was primarily due to light scattering caused by structural damage [93]. In rat lenses, high-glucose exposure decreased levels of GSH, CAT, and AR while increasing RAGE [93]. The models provide a valuable system for testing agents targeting redox balance and AGE formation.

In the organ culture model, exposure to glyceraldehyde significantly elevated MG and MG-derived AGE levels, leading to a sharp decrease in GSH [90]. Inhibition of GLO1 with *S*-[*N*-hydroxy-*N*-(4-chlorophenyl)carbamoyl]glutathione (HCCG) diester further enhanced MG accumulation [90]. Although GSH was expected to detoxify MG via the GLO pathway, experimental data suggested no consistent correlation between GSH levels and MG-AGE accumulation. This inconsistency may reflect the saturation or inhibition of GLO1 activity under severe glycation stress.

Padival and Nagaraj demonstrated the ability of pyridoxamine (PYM) to inhibit AGE formation in diabetic rat lenses [91]. In both in vivo and organ culture settings, PYM reduced levels of argpyrimidine and pentosidine. Furthermore, PYM restored lens GSH levels and increased GLO1 activity, implicating it as a promising therapeutic agent that not only scavenges dicarbonyl precursors but also enhances endogenous antioxidant defense.

Abdelkader et al. investigated the anticataractogenic mechanisms of carnosine, a naturally occurring dipeptide, in porcine and human lens models [92]. Their in vitro and ex vivo assays showed potent antiglycating properties but weak direct antioxidant and metal-chelating effects. Carnosine inhibited AGE formation induced by galactose in porcine lenses and maintained sulfhydryl group integrity in bovine lens homogenates.

Nandi et al. developed carboxitin, a compound that combines GSH diester with mercaptoethylguanidine, to target AGE-induced protein crosslinking in mouse lenses [94]. Carboxitin replenished GSH levels and inhibited glycation-mediated protein aggregation and lens stiffening. It blocked ascorbate-derived AGE precursors such as 3-deoxythreosone (3-DG), supporting its dual action as a GSH booster and dicarbonyl scavenger.

Overall, these findings suggest that GSH, PYM, carnosine, and carboxitin may help restore antioxidant capacity and attenuate glycation-related damage in eye tissues.

### 4.3. In Vivo Studies

Table 5 presents in vivo studies evaluating the effects of AGEs and interventions in models of diabetic and age-related eye diseases. Numerous animal studies have explored the complex relationship between glycation stress and GSH depletion, highlighting the pathophysiological mechanisms and testing diverse therapeutic agents.

Dietary calorie restriction in Emory mice significantly extended the median lifespan by 40% and delayed cataract progression compared to control-fed animals [97]. It reduced plasma glucose and glycohemoglobin levels, enhanced liver GSH levels, and decreased markers of oxidative stress and glycation, suggesting that the suppression of oxidative and glycation damage contributes to a slowdown in cataract development and other age-associated changes [97].

A 5% or 25% galactose diet administered to pigs resulted in polyol accumulation, a slight depletion of GSH, and increased protein glycation in the lens [95]. While a 5% galactose diet induced only initial cataractogenic changes, the 25% diet caused significant lens damage characterized by inositol loss and enhanced protein glycation. In galactosemic rats, pentosidine accumulation linked to oxidative stress was suppressed by an AR inhibitor, i.e., sorbinil, preventing DC formation [96].

In a chemically (with alloxane) induced diabetes model, lipid peroxidation was elevated while antioxidant enzyme activities (SOD and GPx) and nitric oxide production were diminished [98]. Melatonin supplementation restored these parameters to near-normal levels independent of blood glucose control, suggesting its potent antioxidant role [98].

In streptozotocin (STZ)-induced diabetic rats, early insulin treatment significantly improved hyperglycemia, lipid abnormalities, glycosylated hemoglobin levels, oxidative stress markers, advanced glycation end-product accumulation, and the ratio of GSH to GSSG. Early intervention prevented the development of sensory neuropathy and diabetic cataracts (DCs), whereas late insulin treatment failed to show these protective effects [103]. Additionally, vitamin K1 administration in diabetic rats partially reduced hyperglycemia and normalized lens levels of sorbitol, AGEs, calcium, Ca^2+^-ATPase activity, and oxidative stress markers [105]. These effects contributed to the inhibition of DC progression, suggesting vitamin K1 as a promising therapeutic agent.

PYM treatment in STZ-induced diabetic rats inhibited the formation of major AGEs such as argpyrimidine and pentosidine in lenses, possibly by scavenging RCS and enhancing AR activity [91]. Although PM decreased AGE accumulation, it did not fully prevent DC development. In hSVCT2 transgenic mice characterized by ascorbate-induced lenticular glycation, nucleophilic compounds, NC-I (i.e., arginine) and NC-II, but not aminoguanidine (AGD), PYM, or penicillamine, reduced AGE levels such as pentosidine, CML, and CEL [75].

Aspirin has been shown to exert antioxidant effects through the inhibition of NF-κB and reduction in AGE formation [100]. Topical administration of carnosine, AGD, or aspirin in diabetic rats delayed DC progression during early stages by inhibiting AGE accumulation and preserving antioxidant enzymes like CAT and GR [100]. However, their protective effect diminished with severe hyperglycemia, suggesting that excessive glycation overwhelms pharmacological interventions at later stages.

Silica-based CeCl_3_ nanoparticles and *S*-allylmercapto-*N*-acetylcysteine significantly restored lens GSH levels and inhibited AGE-induced oxidative stress and protein aggregation [86,106]. Cemtirestat [113], an AR inhibitor, improved GSH redox status in diabetic eyes.

Natural products like nigerloxin (a fungal metabolite) [101,104] and plant-derived compounds, such as curcumin, astaxanthin, and chrysin [99,109,110], displayed protective effects by modulating antioxidant enzyme activities and maintaining redox balance. Berberine and morin were effective in mitigating AGE-induced damage in DC and DR models [111,112]. Moscatilin inhibited the MAPK/NF-κB pathway and inflammation in Müller cells [88]. *Rosa damascena* hydrosol and extracts of *Anemarrhena asphodeloides* and Zhujing pill reduced malondialdehyde (MDA) levels, restored GSH or CAT activity, and suppressed AGE accumulation, thereby preserving lens structure and function [102,107,108].

Animal models, particularly galactose-fed and STZ-induced diabetic rats, have provided significant insights into the relationship between AGEs, oxidative stress, and GSH metabolism in the lens and retina. The collected animal studies provide robust evidence that glycation-induced oxidative stress plays a central role in the pathogenesis of ocular diseases, particularly through the depletion of GSH and disruption of redox balance. Interventions using natural antioxidants, plant-based compounds, synthetic molecules, and nanomaterials have shown promise in preserving or restoring GSH levels and preventing glycation-mediated lens and retinal damage. These findings strongly support the therapeutic relevance of targeting glycation and redox imbalance to prevent or treat DC, DR, and other ocular complications.

### 4.4. Clinical Studies

Table 6 summarizes human clinical studies analyzing the effects of AGEs and interventions in ocular conditions.

Ahmed et al. demonstrated that CEL accumulates with age in human lens proteins, causing age-related cataracts [114]. In Eales’ disease, with an inflammatory retinal condition, GSH and GPx levels were significantly reduced in both active and healed phases, indicating a chronic redox imbalance [115]. Hashim and Zarina reported that lenses from DC patients showed significantly lower levels of GSH, GPx, GR, and G6PDH compared to non-diabetic cataract patients [116]. In contrast, AR and SDH activities were increased, indicating polyol pathway activation and oxidative imbalance.

Géhl et al. analyzed vitreous humor from patients with proliferative DR and found elevated levels of AGEs and protein carbonyls, confirming oxidative stress [118]. Interestingly, GSH levels were also higher in diabetic samples, possibly reflecting a compensatory antioxidant response to increased oxidative burden. Mynampati et al. examined clear, cortical, and nuclear cataract lenses [119]. They found a significant decrease in GSH in nuclear cataract lenses (a form of age-related cataracts) along with a marked increase in the AGE argpyrimidine, supporting the role of AGEs in promoting redox imbalance and lens opacity.

Babizhayev et al. explored the role of oxidative stress and genetic polymorphisms in antioxidant enzymes in the development of diabetic neuropathy (DN) in type 1 diabetic patients [117]. The study assessed gene variants in CAT, GPx, and GSTs, and found that DN patients exhibited lower levels of GSH and decreased GPx activity compared to those without DN. Specifically, the -262T/T genotype of the CAT gene was associated with higher CAT activity and lower susceptibility to DN. In vitro experiments showed that glycation reactions involving MG generated free radicals, including superoxide, contributing to oxidative stress. The application of SOD-mimetic peptides significantly reduced radical generation, confirming the antioxidant pathway’s protective role. The authors suggested that antioxidant enzyme genotyping may help identify individuals at higher risk for DN and that peptide-based antioxidants may offer a preventive therapeutic approach. Supplementation with antioxidants such as vitamin C and E demonstrated improvements in oxidative defense, while GSH levels varied depending on the disease stage and tissue type [115,120].

Overall, clinical evidence supports the involvement of AGE accumulation and oxidative stress in both age-related and diabetic ocular conditions. However, it is noteworthy that most clinical studies referenced in this review involved relatively small patient cohorts (typically fewer than 100 participants), which may limit the generalizability of the findings.

## 5. Discussion

### 5.1. Mechanistic Insights into GSH Depletion and Redox Imbalance in Glycation-Related Eye Diseases

The eye is vulnerable to oxidative and glycation stress due to its high oxygen consumption, continuous exposure to light, and accumulation of long-lived proteins in avascular tissues [121]. Anatomically, ocular structures such as the lens, retina, and vitreous humor are highly susceptible to oxidative insults and glycation-related damage owing to their limited regenerative capacity and low antioxidant enzyme turnover [122]. The avascular nature of the lens and avascular zones of the retina render these compartments especially dependent on cellular redox systems, and GSH homeostasis is increasingly recognized as a key dynamic biomarker in glycation-related ocular diseases.

**Figure 2:**
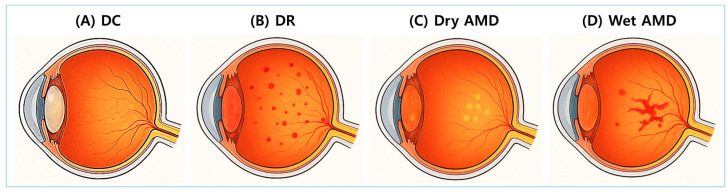
Anatomical illustrations of major glycation-related eye diseases. Cross-sectional images of the eye illustrate four major ocular complications associated with glycation and oxidative stress. (**A**) Diabetic cataracts (DCs): A condition characterized by opacification of the lens. (**B**) Diabetic retinopathy (DR): A diabetes-related complication involving progressive retinal vascular damage. (**C**) Dry age-related macular degeneration (AMD): A condition characterized by the accumulation of drusen and atrophy of the retinal pigment epithelium (RPE). (**D**) Wet AMD: A condition marked by abnormal neovascularization beneath the retina and within the subretinal space.

DC, DR, and AMD are representative glycation-related eye diseases. Figure 2 provides anatomical illustrations of DC, DR, dry AMD, and wet AMD, highlighting distinct damage sites and pathological differences.

In DC, the lens is primarily affected. The lens contains crystallin proteins that must remain transparent and functionally stable throughout life. These proteins are long-lived and exposed to chronic oxidative and glycation stress, especially under hyperglycemic conditions [123]. The lens epithelium also highly expresses glucose transporters and AR, facilitating flux through the polyol pathway [124]. Activation of the polyol pathway in the lens epithelium leads to the accumulation of sorbitol, which induces osmotic stress and promotes the glycation of crystallin proteins [73,116]. This process is associated with a marked decline in GSH levels and reduced activities of antioxidant enzymes such as GPx and GR, resulting in an elevated GSSG/total GSH ratio [74,78,84,85,119]. Clinically, these biochemical alterations correlate with increased lens opacity and cataract severity, particularly in aging and diabetic populations [78,116,119].

The retina is characterized by intense metabolic activity, rich mitochondrial density, and high oxygen tension, making it a prime target for ROS and AGE accumulation [125]. The outer retina, including photoreceptors and the retinal pigment epithelium (RPE), continuously metabolizes visual cycle by-products and polyunsaturated fatty acids (PUFAs) susceptible to oxidative and glycoxidative damage [126]. A key pathological axis links glycation-induced oxidative stress with pathological angiogenesis: GSH depletion aggravates redox imbalance and activates pro-angiogenic signaling pathways, including NF-κB and MAPK, ultimately upregulating vascular endothelial growth factor (VEGF) expression [87,88]. VEGF functions as both a marker and a mediator of this pathological neovascularization [127]. In DR and wet AMD, angiogenesis is primarily driven by sustained redox imbalance and RAGE signaling, both of which are induced by chronic oxidative and glycation stress. While neovascularization in DR originates from the retinal vasculature and leads to vitreous hemorrhage and fibrovascular proliferation, angiogenesis in wet AMD arises from the choroid, resulting in macular disruption, subretinal fluid accumulation, and rapid central vision loss [128,129].

In DR, retinal vasculature cells, particularly endothelial cells, pericytes, and Müller cells, are damaged. This condition is particularly associated with AGE accumulation in retinal capillaries, leading to pericyte loss, vascular leakage, and neovascularization [130]. Chronic hyperglycemia induces the accumulation of AGEs, which activate RAGE signaling and promote the production of ROS and pro-inflammatory cytokines [76,79,80,85,87,88]. This pathological cascade contributes to GSH depletion in retinal endothelial and Müller cells, along with decreased activities of SOD and GPx, thereby exacerbating oxidative damage [76,79,85]. GSH status in the retina has been proposed as a biomarker for both oxidative stress and inflammatory progression in DR and is a promising target for antioxidant-based therapeutic intervention [80,85,87,88,117].

AMD is a progressive neurodegenerative disorder of the macula that is characterized by chronic inflammation, mitochondrial dysfunction, and progressive loss of photoreceptors [131]. It is a leading cause of vision loss in the elderly and exists in two major forms—dry (non-neovascular) and wet (neovascular)—which differ in their pathological mechanisms and clinical progression [132]. Dry AMD is primarily characterized by the accumulation of drusen—extracellular deposits composed of lipids, proteins, and AGEs—between the RPE and Bruch’s membrane [132]. This form of AMD is marked by RPE atrophy and slow photoreceptor loss, predominantly affecting the central macula. In contrast, wet AMD represents an advanced stage of the disease characterized by the pathological upregulation of VEGF, which promotes the formation of choroidal neovascular membranes. These aberrant vessels breach Bruch’s membrane and invade the subretinal space, resulting in fluid leakage, hemorrhage, and rapid central vision loss.

### 5.2. Therapeutic Strategies to Restore GSH Homeostasis Under Glycation Stress

The consistent association between GSH depletion, oxidative stress, and AGE accumulation reinforces the rationale for therapeutic strategies that aim to restore GSH levels and enhance endogenous redox capacity. These strategies are categorized into four mechanistic domains: (I) promoting GSH supply and synthesis, (II) enhancing GSH recycling, (III) mitigating glycation stress, and (IV) reducing oxidative and nitrosative stress. Most of these strategies have been experimentally explored with different approaches. Key findings summarized below support the therapeutic efficacy of these strategies in preserving GSH homeostasis under glycation-induced oxidative stress.

#### 5.2.1. GSH Supply and Synthesis

- Approach 1: Supplementation with GSH precursors

Cysteine is the rate-limiting substrate for intracellular GSH biosynthesis, as its availability directly regulates the activity of GCL, the rate-limiting enzyme in the GSH synthetic pathway [52]. *N*-acetylcysteine and related thiol donors, such as *S*-allylmercapto-*N*-acetylcysteine, increased intracellular GSH levels and reduced ROS in AGE-stimulated ocular and non-ocular cells [86].

- Approach 2: Exogenous GSH administration

Direct supplementation with GSH bypasses the need for de novo synthesis and rapidly increases intracellular GSH levels [133,134]. Exogenously administered GSH inhibited AGE-induced crosslinking in lens proteins [73]. *S*-acetylated GSH analogs improved GSH levels in various experimental systems and showed protective effects against ROS and apoptosis [86].

- Approach 3: Activation of the Nrf2 pathway

Nrf2 activation leads to the transcriptional upregulation of antioxidant response element (ARE)-driven genes, including GCL, GS, and GST [52]. Polyphenolic compounds, such as (−)-EGCG, phloretin, and shogaol, activated Nrf2 signaling in RPE cells, upregulating antioxidant enzymes and increasing intracellular GSH levels in response to dicarbonyl stress [80].

#### 5.2.2. GSH Recycling

- Approach 4: Activation of GR

GR preserves the GSH/GSSG ratio and suppresses ROS accumulation [58]. Grx (also called TTase) overexpression enhanced GSSG reduction and preserved the GSH/GSSG ratio in AGE-stressed ocular models, whereas knockdown exacerbated redox imbalance and ROS accumulation [85].

- Approach 5: Inhibition of AR

As discussed in Section 2.3, AR hyperactivation depletes NADPH and promotes sorbitol accumulation. Inhibiting AR activity has therefore emerged as a strategy to preserve NADPH for GSH regeneration. AR inhibitors, including novel 1-hydroxypyrazole-based compounds and the natural alkaloid berberine, have been shown to effectively suppress polyol pathway flux, preserve NADPH availability, and mitigate GSH depletion in retinal endothelial cells and Müller cells exposed to AGEs [77,81]. In vivo, agents such as berberine, cemtirestat, and stobadine reduced sorbitol levels, decreased AGE and CML accumulation, and improved GSH/GSSG ratios in diabetic rat models [111,113].

- Approach 6: Activation of the pentose phosphate pathway (PPP)

Activation of this pathway, particularly through G6PDH—the rate-limiting enzyme of the PPP—enhances NADPH generation, which is crucial for GSH recycling [135]. In diabetic retinal models, the upregulation of G6PDH activity increased NADPH levels and restored the antioxidant capacity by improving the GSH/GSSG ratio [136].

#### 5.2.3. Mitigation of Glycation Stress

- Approach 7: Activation of GLO system

While the biochemical mechanism of GLO1 has been described in Section 3.2, its therapeutic efficacy varies depending on tissue context and experimental conditions. For example, PYM was shown to enhance GLO1 activity and restore GSH levels in some diabetic and organ culture models, but the extent of its protective effects varied depending on experimental conditions [91]. Intriguingly, the inhibition of GLO1 using HCCG diester led to paradoxical outcomes—elevated MG levels but reduced MG-derived AGEs—suggesting uncoupling between MG availability and AGE formation under certain contexts [90].

- Approach 8: Inhibition of AGE formation

Trapping RCS, such as GO, MG, and glycolaldehyde, represents an effective strategy to suppress AGE formation and reduce GSH consumption [137,138]. AGD reduced CML and CEL formation in diabetic lens models and preserved antioxidant enzyme activity, although its efficacy varied depending on the glycation context [75,99]. PYM and carnosine significantly reduced the levels of pentosidine and argpyrimidine in diabetic and galactose-exposed lenses, respectively, while also preventing GSH depletion and inhibiting protein aggregation in ex vivo systems [92,100]. Carboxitin exhibited dual antiglycation and antioxidant actions by restoring GSH levels, blocking AGE-mediated protein crosslinking, and reducing lens stiffness in ascorbate-accelerated glycation models [94].

#### 5.2.4. Mitigation of Oxidative/Nitrosative Stress

- Approach 9: Direct scavenging of ROS/RNS

Direct scavengers of ROS and RNS reduce oxidative pressure on the intracellular GSH pool and protect ocular tissues from redox imbalance. By neutralizing highly reactive species such as ^•^OH, O_2_^•^⁻, ^•^NO, and ONOO⁻, these compounds help prevent lipid peroxidation, protein nitration, and mitochondrial dysfunction [139]. Antioxidants such as astaxanthin have effectively restored GSH levels and mitigated AGE-induced oxidative stress in ocular models [109]. Supplementation of vitamins C and E has been shown to improve ocular oxidative defense in clinical studies [115,120].

- Approach 10: Inhibition of ROS/RNS-generating enzymes

Endogenous production of ROS and RNS is enzymatically mediated by key oxidoreductases, including NADPH oxidases (NOX), xanthine oxidase (XO), nitric oxide synthase (NOS), and myeloperoxidase (MPO) [140,141,142,143]. These enzymes are upregulated in response to glycation stress and chronic inflammation, contributing to GSH depletion and oxidative damage [144]. Pharmacological inhibition of these enzymes reduces intracellular oxidant burden, preserves NADPH availability, and supports GSH recycling [145]. While specific inhibitors of these enzymes have shown efficacy in systemic diseases, their applicability in ocular conditions remains underexplored and requires further investigation.

### 5.3. Targeting GSH Homeostasis vs. VEGF

Interventions targeting GSH primarily act at upstream levels by alleviating oxidative and carbonyl stress, restoring redox balance, and enhancing intracellular detoxification mechanisms [146]. In contrast, anti-VEGF therapies act on downstream pathophysiological events, particularly by suppressing abnormal angiogenesis and vascular permeability [147]. Despite their mechanistic differences, emerging evidence suggests a pathological link between GSH depletion and VEGF overexpression. This link has been observed in DR and wet AMD, where chronic oxidative stress and GSH deficiency synergistically drive inflammation and neovascularization [148].

**Figure 3:**
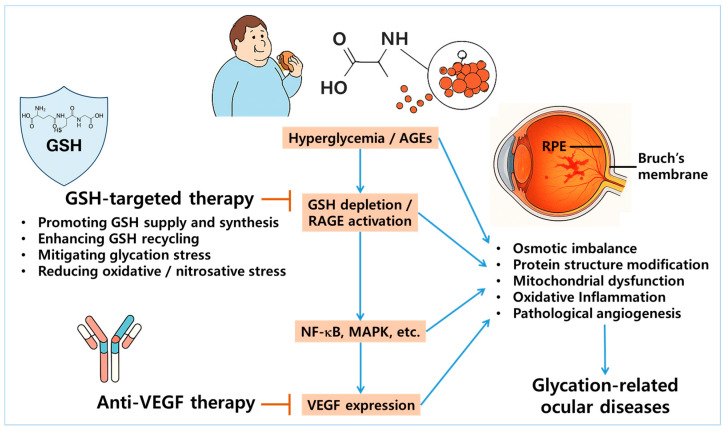
Rationale for dual targeting of GSH homeostasis and VEGF pathways in glycation-related ocular diseases. Chronic hyperglycemia and accumulation of AGEs promote GSH depletion and activate the receptor for AGEs (RAGE), leading to the downstream stimulation of nuclear factor kappa B (NF-κB) and mitogen-activated protein kinase (MAPK) signaling pathways. This cascade ultimately results in the overexpression of vascular endothelial growth factor (VEGF), driving pathological angiogenesis in ocular tissues—particularly within the RPE and Bruch’s membrane. The illustration highlights this mechanistic interplay as a therapeutic rationale for dual targeting: restoring GSH homeostasis to mitigate oxidative and glycation-induced stress, while concurrently inhibiting VEGF to suppress neovascularization. Arrows represent stimulation, whereas blunted bars indicate prevention or inhibition.

Several studies have demonstrated that antioxidant and redox-modulating agents such as morin, cemtirestat, and moscatilin not only restore GSH levels but also suppress VEGF expression in ocular models of glycation stress, indicating that the modulation of GSH homeostasis may indirectly regulate VEGF-driven angiogenic responses [88,112,113]. These findings support the rationale for a dual-targeting strategy that combines redox-based interventions with VEGF inhibition (Figure 3). In advanced stages of DR or wet AMD, where both oxidative damage and neovascularization coexist, such a combined approach may offer synergistic therapeutic benefits [130,132]. Further research is needed to elucidate the cooperative effects of this integrated therapeutic strategy.

### 5.4. Limitations of Current Studies and Future Directions

Despite the encouraging preclinical findings, several limitations are apparent across existing studies. Many investigations have relied predominantly on in vitro or organ culture models, which do not fully replicate the complexity of in vivo ocular environments, including systemic influences and tissue interactions. Significant heterogeneity exists among experimental models, types of glycation inducers, disease stages examined, and intervention protocols, making direct comparisons and generalizations challenging. Clinical translation remains limited; although observational data support the importance of GSH in ocular health, few well-designed clinical trials have specifically evaluated interventions aimed at restoring GSH homeostasis in eye diseases.

Future research should aim to address these gaps by developing targeted ocular delivery systems capable of efficiently delivering GSH precursors, mimetics, or antioxidant molecules to affected tissues. Identifying and optimizing novel Nrf2 activators with improved specificity and safety will be important for enhancing endogenous antioxidant defenses. In parallel, strategies targeting key enzymatic regulators of glycation and redox stress should be further explored. Enhancing GLO system activity may reduce AGE formation while preserving GSH pools, making it a promising therapeutic target in glycation-related ocular damage. Conversely, the inhibition of AR, the first enzyme of the polyol pathway, can prevent excessive NADPH depletion and mitigate downstream glycation and oxidative stress. A deeper mechanistic understanding of the crosstalk between glycation signaling, redox regulation, mitochondrial dysfunction, and inflammation will be critical for refining therapeutic strategies. Large-scale, randomized clinical trials are urgently needed to evaluate the efficacy of GSH-enhancing interventions in preventing or mitigating DC, DR, AMD, and other glycation-related ocular disorders.

Although VEGF inhibitors effectively suppress angiogenesis, they do not address the upstream redox imbalance. GSH-based interventions could act earlier in the pathophysiological cascade, potentially enhancing VEGF inhibitor efficacy and preventing resistance or recurrence. To date, no clinical trials have explicitly tested the combination therapy of VEGF inhibitors with GSH-based interventions in ocular diseases. However, this represents a promising direction for future translational studies.

## 6. Conclusions

GSH homeostasis plays a central role in maintaining redox balance across ocular tissues, with disease-specific patterns of dysregulation contributing to the pathogenesis of glycation-related eye diseases. In DC, GSH depletion is primarily driven by osmotic imbalance and glycation-induced stress within the lens matrix. In DR, chronic oxidative inflammation perturbs GSH regulation in retinal vascular and glial cells. In AMD, mitochondrial dysfunction and angiogenic stress, particularly in the macular region, further compromise GSH-dependent antioxidant defense. Therapeutic strategies aimed at enhancing GSH synthesis, recycling, or preservation have demonstrated promising results in preclinical models. These findings support the rationale for GSH-targeting redox-modulating interventions that could be used either in combination with or as an alternative to anti-VEGF therapy in certain glycation-related ocular disorders.

## Figures and Tables

**Table 1:** Classification of advanced glycation end-products (AGEs) by precursor substrate and specific molecular target sites.

Precursor Substrate	Molecular Target Sites					
	Lysine Residues	Arginine Residues	Cysteine Residues	Collagen	Histone Proteins/DNA	Phospholipids/PUFA
Glucose	CML, CEL			Glucosepane	Histone glycation (CML)	Lipid peroxidation–AGEs
Fructose	FL, CML			Crosslinks	Nucleosomal AGE adducts	
Galactose	CML, CEL	Pentosidin, Argpyrimidine		Galactose crosslinks	Nucleosomal AGEs	
MG	CEL, Argpyrimidine	MG-H1, Argpyrimidine	MG–cysteine	MG crosslinks	MG-dG	MG–phospholipid adducts
GO	CML	GO-H1	GO–thioesters	GO–collagen	GO-dG	GO–lipid adducts
3-DG	3-DG-CML	3-DG-H1		Crosslinks	3-DG–DNA adduct	
Glyceraldehyde	Glycer-AGEs	Glycer-Argpyrimidine		Glycer-crosslinks	DNA oxidation products	
Glycolaldehyde	CML, CEL	Argpyrimidine	Glyco-thioesters	Crosslinks	DNA adducts	
Ribose	Rib-CML, Rib-CEL	Rib-H1, Rib-Argpyrimidine		Ribose crosslinks	Rib–DNA adducts, histone glycation	
Ascorbate (oxidized)	CML, CEL	Argpyrimidine	Thiol oxidation products	Ascorbyl-collagen adducts	DNA strand breaks	Ascorbyl–lipid adducts
Acetoacetate	CEL		Cysteine adducts	Acetoacetate-derived crosslinks		
HNE, MDA	HNE-CML, MDA-CEL		Michael adducts	Collagen stiffening	MDA-dG, HNE–DNA adducts	HNE–phospholipid adducts

Adapted and integrated from previous studies [6,30,31,32,33,34]. The definitions of abbreviations used in this table can be found in the abbreviation list provided separately.

**Table 2:** Glutathione (GSH)-related enzymatic and non-enzymatic reactions.

Reaction Types	Reactions	Enzymes	Functions
Non-enzymatic	GSH + ROS (e.g., ^•^OH, ¹O_2_) ⟶ Oxidation products of GSH + Less reactive products of ROS		Scavenging of ROS
Enzymatic	2 GSH + H_2_O_2_ ⟶ GSSG + 2 H_2_O	GPx	Detoxification of H_2_O_2_ and lipid hydroperoxides
Enzymatic	GSSG + NADPH + H^+^ ⟶ 2 GSH + NADP^+^	GR	Regeneration of GSH
Enzymatic	2GSH + DHA ⟶ GSSG + Ascorbate	Grx	Regeneration of ascorbate
Enzymatic	GSH + Protein-SH ⟶ Protein-SSG	Grx	*S*-glutathionylation and redox signaling
Enzymatic	GSH + Protein-SSG ⟶ GSSG + Protein-SH	Grx	Deglutathionylation and reduction of protein–glutathione mixed disulfides
Enzymatic	2 GSH + Protein-S-S-Protein ⟶ GSSG + 2 Protein-SH	Grx	Reduction of interprotein disulfides
Non-enzymatic	2 GSH + DHA ⟶ Ascorbate + GSSG		Regeneration of ascorbate
Enzymatic	2 GSH + DHA ⟶ Ascorbate + GSSG	Grx	Regeneration of ascorbate
Non-enzymatic	GSH + RNS (e.g., ONOO⁻) ⟶ Oxidation products of GSH + Less reactive products of RNS		Scavenging of RNS
Non-enzymatic	GSH + ^•^NO ⟶ GSNO		Buffering of ^•^NO
Enzymatic	GSNO + NADH + H^+^ ⟶ [GSNHOH] + NAD^+^ [GSNHOH] + GSH ⟶ GSSG + NH_2_OH	GSNOR	Detoxification of GSNO
Enzymatic	GSH + RCS (e.g., Glycolaldehyde) ⟶ GSH conjugates	GST	Detoxification of electrophilic RCS compounds
Non-enzymatic	GSH + MG ⟶ Hemithioacetal GSH + GO ⟶ Hemithioacetal		Initial trapping of reactive aldehydes
Enzymatic	GSH + MG ⟶ *S*-lactoylglutathione GSH + GO ⟶ *S*-glycolylglutathione	GLO1	Detoxification of reactive aldehydes
Enzymatic	*S*-lactoylglutathione + H_2_O ⟶ Lactate + GSH *S*-glycolylglutathione + H_2_O ⟶ Glycolate + GSH	GLO2	Completion of the detoxification cycle of reactive aldehydes

The definitions of abbreviations used in this table can be found in the abbreviation list provided separately.

**Table 3:** Cellular responses to AGEs and effects of different interventions in in vitro models.

Experimental Models	Glycation Inducers	Induced Changes	Interventions	Outcomes or Findings	References
Bovine retinal microvascular endothelial cells and pericytes	a. High glucose b. AGE-BSA	a. GPx activity (↓) in endothelial cells b. CAT (↑), SOD (↑) in pericytes			Paget et al., 1998 [72]
Bovine lens proteins	a. Threose	a. Protein crosslinking (↑)	a. AGD b. Semicarbazide c. Phenylene diamine d. Cysteine e. GSH f. Sodium metabisulfite	a–f. Crosslinking (↓), sodium metabisulfite is most potent	Lehman and Ortwerth, 2001 [73]
Human lens epithelial cells (HLE-B3)	a. High glucose (50–100 mM)	a. GSH (↓), lipid peroxidation (↑), antioxidant enzymes (↓)			Shin et al., 2006 [74]
Calf lens protein homogenates	a. DHA (1 mM) b. Ascorbate (25 mM)	a,b. CML (↑), CEL (↑), pentosidine (↑), fluorescent crosslinks (↑)	a. AGD b. PYM c. Penicillamine d. NC-I e. NC-II	a–e. Fluorescent AGEs (↓), pentosidine (↓), CML (↓), CEL (↓)	Fan and Monnier, 2008 [75]
Porcine retinal pericytes	a. AGEs	a. ROS (↑), apoptosis (↑), SOD (↓), GSH (↓)	a. PEDF	a. ROS (↓), SOD (↑), GSH (↑), apoptosis (↓) via Src pathway	Sheikpranbabu et al., 2011 [76]
In vitro enzyme assay	a. AR pathway (glucose-derived)	a. ROS (↑), GSH (↓)	a. 1-Hydroxypyrazole-based AR inhibitors	a. AR activity (↓), antioxidant profile (↑)	Papastavrou et al., 2013 [77]
Human lens epithelial cells	a. Fructose	a. AGE (↑), ROS (↑), GSH (↓)	a. Silica-based CeCl_3_ nanoparticles b. AGD c. Carnosine	a. AGE (↓), ROS (↓), GSH (↑), better than AGD and carnosine b. AGE (↓) c. AGE (↓)	Yang et al., 2014 [78]
Human retinal endothelial cells (HRECs)	a. GO	a. ROS (↑), GSH (↓), GPx (↓), SOD (↓)	a. PUGNAc b. OGT siRNA	a. ROS (↓), GSH (↑), GPx (↑), SOD (↑), apoptosis (↓) b. ROS (↑), antioxidant defense (↓), apoptosis (↑)	Liu et al., 2015 [79]
Human retinal epithelial cells	a. Dicarbonyl stress (GO, MG)	a. ROS (↑), GSH (↓), MDA (↑)	a. (−)-EGCG and catechin (green tea); phloretin and phloridzin (apple); 6-shogaol and 6-gingerol (ginger)	a. ROS (↓), GSH (↑), MDA (↓)	Sampath et al., 2016 [80]
Human Müller cells	a. Highly oxidized glycated low-density lipoprotein	a. ROS (↑), apoptosis (↑), GPx (↓), inflammation (↑)	a. Berberine b. Berberine + AMPK inhibitor	a. Oxidative stress (↓), inflammation (↓), apoptosis (↓), AMPK (↑) b. The effects of berberine were blocked by AMPK inhibition	Fu et al., 2016 [81]
Human retinal pigment epithelial cells	a. Light-induced photo-oxidation of A2E (retinal fluorophore)	a. ROS (↑), GSH (↓), MG adducts (↑), RAGE (↑)	a. Quercetin b. Cyanidin-3-glucoside	a. Photo-oxidation (↓), ROS (↓), GSH (↑) b. Similar effects as quercetin	Wang et al., 2017 [82]
In vitro vitreal simulation	a. Riboflavin b. Ascorbate	a. ROS (↑), AGE (↑), protein degradation (↑)	a. GSH	a. Light-induced damage (↓)	Mantha et al., 2020 [83]
Human kidney epithelial cells (HK-2)	a. AGE (100 µg mL^−1^)	a. ROS (↑), GSH (↓), fibrosis markers (↑)	a. Caffeic acid	a. ROS (↓), GSH (↑), fibrosis and EMT markers (↓), via β-catenin pathway	Jeon et al., 2021 [84]
Human lens epithelial cells	a. High glucose b. AGE-BSA	a. ROS (↑), GSH (↓), GSSG to total GSH ratio (↑), SOD, CAT (↓)	a. TTase knockdown	a. Oxidative damage (↑), TTase protects lens cells	Liu et al., 2021 [85]
Rat bone marrow stromal cells (BMSCs)	a. Glyco-BSA b. Rib-BSA c. H_2_O_2_	a-c. Cell survival (↓), ROS (↑)	a. *S*-Allyl mercapto-*N*-acetylcysteine (0.2 mM)	a. Cell survival (↑), ROS (↓)	Abu-Kheit et al., 2022 [86]
Adult retinal pigment epithelial cells (ARPE-19)	a. High glucose (34, 68 mM) b. AGE (with 17–68 mM glucose)	a. NF-κB (↑), VEGF (↑), IL-8, MCP-1, MMP-2 (↑), GPx (↓), PPAR-γ (↓), soluble RAGE (↓) b. Same as a	a. Methanol extract of *Peperomia pellucida* (3 mg mL^−1^) b. Its ethyl acetate fraction (4 mg mL^−1^)	a. NF-κB (↓), pro-inflammatory markers (↓), GPx (↑), PPAR-γ (↑), soluble RAGE (↑) b. Similar to a	Ho et al., 2024 [87]
Mouse retinal Müller cells	a. High glucose (35 mM)	a. Cell viability (↓), ROS (↑), MDA (↑), SOD (↓), CAT (↓), GSH/GSSG (↓), RAGE (↑), TNF-α (↑), IL-1β (↑), IL-6 (↑), MMP-2 (↑), MMP-9 (↑), VEGF (↑), NF-κB (↑), phosphorylation of p38-MAPK and JNK (↑)	a. Moscatilin (0.1–1 μM)	a. Cell viability (↓), oxidative stress (↓), inflammatory markers (↓), NF-κB pathway (↓), p38-MAPK/JNK pathway (↓)	Zhu et al., 2025 [88]

The definitions of abbreviations used in this table can be found in the abbreviation list provided separately. Arrows pointing up (↑) or down (↓) represent increased or decreased levels, respectively.

**Table 4:** Glycation-related changes and intervention outcomes in isolated lens and retinal tissues under ex vivo conditions.

Experimental Models	Glycation Inducers	Induced Changes	Interventions	Outcomes or Findings	References
Rat lens	a. Glyceraldehyde	a. MG (↑), MG-AGEs (↑), GSH (↓)	a. HCCG diester	a. MG (↑), MG-AGEs (↓), partial protection of GSH	Shamsi et al., 2000 [90]
Rat lens	a. High glucose	b. GSH (↓)	a. PYM	a. No effects on GSH	Padival and Nagaraj, 2006 [91]
Human lens epithelial cells and porcine lenses	a. High galactose	a. AGE (↑), GSH (↓), protein aggregation (↑)	a. Carnosine	a. AGE (↓), lens opacification (↓)	Abdelkader et al., 2016 [92]
Human and rat lenses	a. 55 mM glucose	a. GSH (↓), AR (↓), CAT (↓), RAGE (↑), opacity (↑)			Alghamdi et al., 2018 [93]
Mouse lens	a. Erythrulose	a. AGE (↑), protein crosslinking (↑), lens stiffness (↑), GSH (↓)	a. Carboxitin	a. AGE (↓), crosslinking (↓), GSH (↑), mechanical stiffness (↓)	Nandi et al., 2021 [94]

The definitions of abbreviations used in this table can be found in the abbreviation list provided separately. Arrows pointing up (↑) or down (↓) represent increased or decreased levels, respectively.

**Table 5:** In vivo effects of AGEs and therapeutic interventions in animal models.

Experimental Models	Glycation Inducers	Induced Changes	Interventions	Outcomes or Findings	References
Pigs	a. 5% galactose diet b. 25% galactose diet	a. GSH (↓), protein glycation (↑) b. GSH (↓), protein glycation (↑)			Birlouez-Aragon et al., 1989 [95]
Sprague Dawley rats	a. 33% galactose diet	a. GSH (↓), pentosidine (↑)	a. Sorbinil	a. GSH (↑), pentosidine (↓), fluorescence (↓)	Nagaraj et al., 1994 [96]
Emory mice (a strain derived from CFW mice, specifically bred to develop bilateral cataracts)	a. Normal aging	a. Glucose (↑), ascorbate (↓), GSH (↓, 22-month-old mice), glycation (↑)	a. Calorie restriction (40%)	a. Cataract progression (↓), glycohemoglobin (↓), GSH (↑, 22-month-old mice)	Taylor et al., 1995 [97]
Wistar rats	a. Alloxan-induced diabetes	a. GSH (↓), SOD (↓), lipid peroxidation (↑)	a. Melatonin	a. GSH (↑), antioxidant enzyme levels (↑)	Sailaja Devi et al., 2000 [98]
Sprague Dawley rats	a. 30% galactose diet	a. Lipid peroxidation (↑), AGE fluorescence (↑), GSH (↓), protein aggregation (↑)	a. Curcumin 0.002% b. Curcumin 0.01%	a. DC onset/ maturation (↓), lipid peroxidation (↓), AGE (↓) b. Less effective	Suryanarayana et al., 2003 [99]
Sprague Dawley rats	a. STZ-induced diabetes	a. AGEs (pentosidine) (↑), GSH (↓), GLO1 (↓), AR (↑)	a. PYM	a. AGE (argpyrimidine and pentosidine) (↓), GLO1 (↑), AR (↑), no effects on GSH	Padival and Nagaraj, 2006 [91]
hSVCT2 transgenic mice	a. Ascorbate-accelerated aging	a. CML (↑), CEL (↑), pentosidine (↑), fluorescent crosslinks (↑)	a. AGD b. PYM c. Penicillamine d. NC-I e. NC-II	d–e. Pentosidine (↓), fluorescence (↓)	Fan and Monnier, 2008 [75]
Sprague Dawley rats	a. STZ-induced diabetes	a. AGEs (↑), GSH (↓), CAT (↓), GR (↓)	a. Carnosine drops b. AGD drops c. Aspirin drops	a–c. AGE (↓), GSH (↑), enzyme activity (↑), DC progression (↓, carnosine most effective)	Yan et al., 2008 [100]
Wistar rats	a. STZ-induced diabetes	a. AGEs (↑), GSH (↓), SOD, GST, GPx (↓), AR (↑), SDH (↑)	a. Nigerloxin (fungal metabolite)	a. AGE (↓), antioxidant enzymes (↑), polyol pathway enzymes (↓), DC (↓)	Suresha et al., 2012 [101]
Wistar rats	a. STZ-induced diabetes	a. AGE (↑), sorbitol (↑), MDA (↑), SOD (↓), GPx (↓)	a. Ethanol extract of the rhizome of *Anemarrhena asphodeloides*	a. AGE (↓), MDA (↓), SOD (↑), GPx (↑), sorbitol (↓), retinal and lens structure (↑)	Li et al., 2013 [102]
Wistar rats	a. STZ-induced diabetes	a. DC (↑), lens opacity (↑), oxidative stress (↑)	a. Early insulin treatment	a. DC (↓), antioxidant defense (↑)	Balakumar et al., 2013 [103]
Wistar rats	a. Galactose diet	a. AGE (↑), lipid peroxides (↑), GSH (↓), SOD (↓), GPx (↓)	a. Nigerloxin (fungal metabolite)	a. AGE (↓), lipid peroxides (↓), antioxidant enzymes ↑	Suresha and Srinivasan, 2013 [104]
Wistar rats	a. STZ-induced diabetes	a. AGE (↑), sorbitol (↑), GSH (↓), Ca^2+^ (↑), Ca^2+^-ATPase (↓)	a. Vitamin K1	a. AGE (↓), sorbitol (↓), GSH (↑), Ca^2+^-ATPase (↑), DC (↓)	Sai Varsha et al., 2014 [105]
Wistar rats	a. STZ-induced diabetes	a. AGE (↑), lipid peroxidation (↑), protein carbonylation (↑), GSH (↓)	a. Silica-based CeCl_3_ nanoparticles	a. AGE (↓), lipid peroxidation (↓), GSH (↑), DC (↓)	Yang et al., 2017 [106]
Sprague Dawley rats	a. STZ-induced diabetes	a. AGE (↑), SOD (↓), GPx (↓), inflammation markers (↑)	a. Zhujing pill extract	a. AGE (↓), inflammatory markers (↓), antioxidant enzyme levels (↑)	Lei et al., 2018 [107]
Sprague Dawley rats	a. STZ-induced diabetes	a. AGE (↑), AR (↑), GPx (↓), GSH (↓), DC signs (↑)	a. *Rosa damascena* hydrosol (two concentrations)	a. AGE (↓), AR (↓), GPx (↑), GSH (↑), lens structure (↑)	Demirbolat et al., 2019 [108]
Sprague Dawley rats	a. STZ-induced diabetes	a. AGE (↑), MDA (↑), GSH (↓), SOD (↓), CAT (↓)	a. Astaxanthin (low dose) b. Astaxanthin (high dose)	a. AGE (↓), MDA (↓), GSH (↑), SOD (↑), CAT (↑), DC (↓) b. Similar antioxidant effects, dose-responsive trend	Yang et al., 2020 [109]
Wistar rats	a. STZ-induced diabetes	a. Oxidative stress (↑), GSH (↑), lipid/protein damage (↑)	a. Chrysin (50 mg kg^−1^) b. Chrysin (100 mg kg^−1^)	a. GSH (↑), oxidative damage (↓) b. Similar trend, no effect on AGEs or polyol pathway	Wojnar et al., 2020 [110]
Wistar rats	a. STZ-induced diabetes	a. AGEs (↑), AR (↑), AOPP (↑), GSH (↓), SOD (↓), CAT (↓), GPx (↓)	a. Berberine (50 mg/kg)	a. AGEs (↓), AR (↓), AOPP (↓), antioxidant enzymes (↑), GSH (↑)	Zych et al., 2020 [111]
Wistar rats	a. STZ-induced diabetes	a. ROS (↑), MAD (↑), GPx (↓), CAT (↓), SOD (↓), VEGF (↑), TNF-α (↑)	a. Morin (25 mg kg^−1^) b. Morin (50 mg kg^−1^) c. Metformin (350 mg kg^−1^)	a. Oxidative stress (↓), VEGF (↓), TNF-α (↓) b. Greater effect than a. c. Standard comparison	Jiang et al., 2020 [112]
Homozygote C57BLKS/J Leprdb^+/+^ female mice (db/db; diabetic)	a. AGEs	a. ROS (↑), GSH (↓), cell damage (↑)	a. *S*-allyl mercapto-*N*-acetylcysteine (50 mg kg^−1^ day^−1^)	a. GSH (↑), ROS (↑), bone health (↑)	Abu-Kheit et al., 2022 [86]
Wistar rats	a. High-fructose diet b. High fructose + STZ	a. GSH/GSSG ratio (↓), GST (↓), TNF-α (↑), IL-1β (↑), NF-κB (↑), caspase-3 (↑), VEGF (↑), CML (↑), sorbitol (↑) b. More pronounced changes than a	a. Cemtirestat (2.5, 7.5 mg kg^−1^) b. Epalrestat (25, 50 mg kg^−1^) c. Stobadine (25, 50 mg kg^−1^)	a–c. TNF-α (↓), IL-1β (↓), NF-κB (↓), caspase-3 (↓), sorbitol (↓) a, c. GSH/GSSG ratio (↑), GST (↑), CML (↓) a, b. Sorbitol (↓) b. VEGF (↓)	Reihanifar et al., 2023 [113]
DR mice	a. STZ + high-fat diet	a. GFAP(↓), vimentin (↓), ROS (↑), MDA (↑), SOD (↓), CAT (↓), TNF-α (↑), IL-1β (↑), IL-6 (↑), VEGF (↑), NF-κB (↑), phosphorylation of p38-MAPK and JNK (↑)	a. Moscatilin (25 mg kg^−1^)	a. Retinal structure (↑), oxidative stress (↓), inflammatory markers (↓), NF-κB pathway (↓), p38-MAPK/JNK pathway (↓)	Zhu et al., 2025 [88]

The definitions of abbreviations used in this table can be found in the abbreviation list provided separately. Arrows pointing up (↑) or down (↓) represent increased or decreased levels, respectively.

**Table 6:** Clinical studies on the effects of AGEs and therapeutic interventions in human subjects.

Study Model and Format	Glycation Inducers	Induced Changes	Interventions	Outcomes or Findings	References
Human donor lenses (*n* = 12); 8 aged, 4 young	a. Normal aging	a. CEL (↑)			Ahmed et al., 1997 [114]
Eales’ disease patients (*n* = 22); case–control observational	a. Oxidative and glycoxidative stress	a. GSH (↓), SOD (↓), lipid peroxidation (↑), CML (↑)	a. Vitamins E and C b. Methotrexate (low dose)	a. Antioxidant markers (↑) b. Clinical symptoms (↓)	Ramakrishnan et al., 2007 [115]
Human lenses from diabetic patients (*n* = 20)	a. Osmotic stress and chronic hyperglycemia	a. GSH (↓), AR (↑), SDH (↑), AGEs (↑)			Hashim and Zarina, 2012 [116]
Human type 1 diabetic patients (*n* = 70); randomized, double-blind clinical trial	a. Chronic hyperglycemia	a. ROS (↑), GSH (↓), antioxidant gene variation (↑ susceptibility)	a. Imidazole-based peptide antioxidants	a. ROS (↓), GSH (↑), antioxidant enzyme (↑), neuropathy risk (↓)	Babizhayev et al., 2015 [117]
Vitreous humor from diabetic donors (*n* = 40)	a. Diabetes (hyperglycemia)	a. AGEs (↑), protein carbonylation (↑), GSH (↑)			Gehl et al., 2016 [118]
Cataractous lenses (*n* = 60) and clear lenses (*n* = 15)	a. Age-related glycation	a. GSH (↓ in nuclear > cortical), argpyrimidine (↑ in nuclear)			Mynampati et al., 2017 [119]

The definitions of abbreviations used in this table can be found in the abbreviation list provided separately. Arrows pointing up (↑) or down (↓) represent increased or decreased levels, respectively.

## Data Availability

The original contributions presented in this study are included in the article, and further inquiries can be directed to the corresponding author.

## Abbreviations

3-DG	3-deoxyglucosone
A2E	*N*-retinylidene-*N*-retinylethanolamine
AGD	aminoguanidine
AGE	advanced glycation end-product
AGE-R	advanced glycation end-product receptor
AKR	aldo-keto reductase
ALDH	aldehyde dehydrogenase
AMD	age-related macular degeneration
AMPK	adenosine monophosphate-activated protein kinase
AOPP	advanced oxidation protein product
AR	aldose reductase
ARE	antioxidant response element
BSA	bovine serum albumin
CAT	catalase
CEL	*N*^ε^-(carboxyethyl)lysine
ChaC	glutathione-specific γ-glutamylcyclotransferase
CML	*N*^ε^-(carboxymethyl)lysine
DCs	diabetic cataracts
dG	deoxyguanosine
DHA	dehydroascorbate
DN	diabetic neuropathy
DNA	deoxyribonucleic acid
DR	diabetic retinopathy
EMT	epithelial–mesenchymal transition
EGCG	epigallocatechin gallate
ER	endoplasmic reticulum
FL	fructoselysine
FR3K	fructosamine-3-kinase
G6PDH	glucose-6-phosphate dehydrogenase
GCL	γ-glutamylcysteine ligase
GFAP	glial fibrillary acidic protein
GGT	γ-glutamyl transpeptidase
GGCT	γ-glutamylcyclotransferase
GLO	glyoxalase
GO	glyoxal
GPx	glutathione peroxidase
GR	glutathione reductase
Grx	glutaredoxin
GS	glutathione synthetase
GSH	glutathione
GSNOR	S-nitrosoglutathione reductase
GSSG	glutathione disulfide
GST	glutathione S-transferase
H1	hydroimidazolone-1
HO-1	heme oxygenase-1
HCCG	*S*-[*N*-hydroxy-N-(4-chlorophenyl)carbamoyl]glutathione
IL	interleukin
JNK	c-Jun *N*-terminal kinase
MAPK	mitogen-activated protein kinase
MCP	monocyte chemoattractant protein
MDA	malondialdehyde
MG	methylglyoxal
MMP	matrix metalloproteinase
MPO	myeloperoxidase
MRP	multidrug resistance-associated protein
NC	nucleophilic compound
NF-κB	nuclear factor kappa-light-chain-enhancer of activated B cells
NOS	nitric oxide synthase
NOX	NADPH oxidase
Nrf2	nuclear factor erythroid 2-related factor 2
OGA	*O*-linked *N*-acetylglucosaminidase
OGT	*O*-linked *N*-acetylglucosamine transferase
PARK	Parkinsonism-associated deglycase
PEDF	pigment epithelium-derived factor
PPAR-γ	peroxisome proliferator-activated receptor gamma
PPP	pentose phosphate pathway
PUFA	polyunsaturated fatty acid
PUGNAc	*O*-(2-Acetamido-2-deoxy-D-glucopyranosylidene)amino-*N*-phenylcarbamate
PYM	pyridoxamine
Prx	peroxiredoxin
RAGE	receptor for advanced glycation end-products
RCS	reactive carbonyl species
RNS	reactive nitrogen species
ROS	reactive oxygen species
RPE	retinal pigment epithelium
SDH	sorbitol dehydrogenase
siRNA	small interfering RNA
SOD	superoxide dismutase
STZ	streptozotocin
TNF	tumor necrosis factor
TTase	thioltransferase
VEGF	vascular endothelial growth factor
XO	xanthine oxidase
